# Computational models for prediction of protein–protein interaction in rice and *Magnaporthe grisea*

**DOI:** 10.3389/fpls.2022.1046209

**Published:** 2023-02-01

**Authors:** Biswajit Karan, Satyajit Mahapatra, Sitanshu Sekhar Sahu, Dev Mani Pandey, Sumit Chakravarty

**Affiliations:** ^1^Department of Electronics and Communication Engineering, Birla Institute of Technology, Ranchi, India; ^2^Department of Bioengineering and Biotechnology, Birla Institute of Technology, Ranchi, India; ^3^Department of Electrical and Computer Engineering, Kennesaw State University, Kennesaw, GA, United States

**Keywords:** rice, *M. grisea*, interolog, domain, gene ontology, phylogenetic, SVM

## Abstract

**Introduction:**

Plant–microbe interactions play a vital role in the development of strategies to manage pathogen-induced destructive diseases that cause enormous crop losses every year. Rice blast is one of the severe diseases to rice *Oryza sativa* (*O. sativa*) due to *Magnaporthe grisea* (*M. grisea*) fungus. Protein–protein interaction (PPI) between rice and fungus plays a key role in causing rice blast disease.

**Methods:**

In this paper, four genomic information-based models such as (i) the interolog, (ii) the domain, (iii) the gene ontology, and (iv) the phylogenetic-based model are developed for predicting the interaction between *O. sativa* and *M. grisea* in a whole-genome scale.

**Results and Discussion:**

A total of 59,430 interacting pairs between 1,801 rice proteins and 135 blast fungus proteins are obtained from the four models. Furthermore, a machine learning model is developed to assess the predicted interactions. Using composition-based amino acid composition (AAC) and conjoint triad (CT) features, an accuracy of 88% and 89% is achieved, respectively. When tested on the experimental dataset, the CT feature provides the highest accuracy of 95%. Furthermore, the specificity of the model is verified with other pathogen–host datasets where less accuracy is obtained, which confirmed that the model is specific to *O. sativa* and *M. grisea.* Understanding the molecular processes behind rice resistance to blast fungus begins with the identification of PPIs, and these predicted PPIs will be useful for drug design in the plant science community.

## 1 Introduction

Rice (*Oryza sativa*) is an important crop, and its production is affected by several abiotic and biotic stresses. Among biotic stresses, the *Magnaporthe grisea* (*M. grisea*) fungus is the most harmful and causes a loss of 30%–40% in yield that is enough to feed millions of people (Parker et al., 2008). Blast fungus can affect the rice plant parts like leaves, roots, panicles, and nodes during its growth period. In addition, the blast fungus is detrimental to small grains like wheat and results in a significant reduction in yield (Dean et al., 2005; Xue et al., 2012). One of the most efficient and economical means for controlling the fungal diseases is by increasing the potential of resistance in the host plant. For these diseases, genetic engineering has been a successful and cost-effective approach in the last few decades (Hulbert et al., 2001; Ribot et al., 2008). The experimental detection of protein–protein interactions (PPIs) between plant and pathogen is a cumbersome process. Until now, few numbers have been reported of experimental PPIs between *O. sativa* and *M. grisea* that are inadequate to explore the pathogenic molecular mechanism (Pellegrini et al., 1999; Jia et al. 2000; Krogh et al., 2001; Ng et al., 2003; Salwinski et al., 2004; Quevillon et al., 2005; Shoemaker and Panchenko, 2007; Wang et al., 2007; Najafabadi and Salavati, 2008; Parker et al., 2008; Ribot et al., 2008; Kumar and Nanduri, 2010; Mukhtar et al., 2011; Li et al., 2012; Maetschke et al., 2012; Mentlak et al., 2012; Park et al., 2012; Schleker et al., 2012; Simonsen et al., 2012; Meyer et al., 2013; Mosca et al., 2014; Rao et al., 2014; Sahu et al., 2014; Tully et al., 2014; Nourani et al., 2015; Li et al., 2016; Singh et al., 2016; Klopfenstein et al., 2018; Savojardo et al., 2018; Karan et al., 2019; Ma et al., 2019; Sahu et al., 2019; Loaiza et al., 2020; Lu et al., 2020; Singh et al., 2020; Wang et al., 2020; Rapposelli et al., 2021; Kumar et al., 2022; Mishra et al., 2022; Wu et al., 2015). Therefore, the computational approach is seen as an alternative method for the large-scale identification of PPIs. The computational approaches for PPI prediction include genomic data-based predictor (Barker and Pagel, 2005; Najafabadi and Salavati, 2008), protein structure (Aloy and Russell, 2002), domain details (Huang et al., 2007; Wang et al., 2007), protein sequence (Zhang et al., 2012), and semantic similarity of gene ontology (GO) annotations (Maetschke et al., 2012). The majority of these algorithms are based on data mining, which uses information from existing PPIs to predict new interactions (Jaeger et al., 2008). Among these computational methods, the interolog and domain-based methods (Wu et al., 2006; Shoemaker and Panchenko 2007; Wang et al., 2007; Simonsen et al., 2012; Tully et al., 2014; Wu et al., 2015; Singh et al., 2016; Singh et al., 2020; Wang et al., 2020; Wuchty, (2011)) are extensively used methodologies for the prediction of PPIs. The potential PPIs between *Homo sapiens* (*H. sapiens*) and *Plasmodium falciparum* (*P. falciparum*) are predicted previously (Dyer et al., 2007) using domain information of the host–pathogen system. Recently interolog and domain-based information to obtain PPIs between *B. pseudomalei* and human has been utilized (Loaiza et al., 2020). An earlier GO-based model was presented for yeast protein interaction (Wu et al., 2006). (Zhou et al., 2013) used domain information of *H. sapiens* and *M. tuberculosis* to obtain the PPIs. (Zhu et al., 2011) have obtained 76,585 PPIs by involving 5,049 rice proteins. Previously, a prediction network based on rice blast fungus was also established (He et al., 2008). In the present study, the authors have predicted 11,674 interactions involving 3,017 blast fungus proteins using an interolog-based approach. From different literature, it was found that computational efforts have hardly been utilized for predicting interspecies PPIs between *O. sativa* and *M. grisea*. A computer-based approach has been created for discovering known *Arabidopsis thaliana* PPIs and to find new PPIs on a genome-scale (Ding and Daisuke , 2019). Ma et al. 2019) have predicted the PPI networks between rice and *M. grisea* using the interolog and domain-based method. However, the method was not implemented at the genome scale. Also, the developed machine learning model was neither tested with the independent experimental dataset nor was it validated with another pathogen–host system to check its reliability.

In this paper, four computational models, the interolog, domain-based, GO, and phylogenetic prediction approaches, are developed to predict the PPIs on a genome-wide scale between rice and *M. grisea*. The high confident PPIs are obtained by intersecting all four computational methods. In the present study, a well-analyzed filtering method has been proposed to identify the potential candidate proteins for interactions. Additionally, a machine learning model using support vector machine has been developed to predict the PPIs efficiently between rice and *M. grisea.*

## 2 Materials and methods

### 2.1 Retrieval of protein sequences

A total of 11,054 protein sequences of *M. grisea* (blast fungus) genome were collected from the Broad Institute website (http://www.broadinstitute.org/annotation/genome/magnaporthe_grisea/MultiHome.html). Similarly, 66,153 protein sequences of rice genome were collected from the MSU database (ftp://ftp.plantbiology.msu.edu/pub/data/Eukaryotic_Projects/o_sativa/annotation_dbs/pseudomolecules/version_7.0/all.dir/).

### 2.2 Filtering of rice proteins to obtain positive-like candidate proteins

In this study, a new approach based on keyword filtering was used to obtain the probable interacting rice proteins. From different kinds of literature surveys [6-15], a set of keywords (**Supplementary Tables 1 and 2**) related to intraspecies and interspecies were retrieved. Another set of keywords (**Supplementary Table 3**) were obtained from plantTFDB v 5.0 (http://planttfdb.cbi.pku.edu.cn/). These keywords are related to the transcription factor of rice and utilized to filter positive-like candidates from the whole-genome rice sequence. The keywords present in rice protein annotation were filtered out as positive candidates. These filtered protein sequences are likely to participate in the interaction. From the above filtering process, only 3,665 rice proteins are extracted. To get homologs of 3,665 proteins, these are subjected to blast analysis against the remaining 62,488 rice proteins having an E-value of 10^-5^. From this analysis, a total of only 8,426 homolog proteins were also obtained. Thus, a cumulative total of 12,091 (3,665 + 8,426) positive-like rice proteins were obtained that might participate in the interactions. On the other hand, the remaining 54,062 proteins that do not participate in the interaction were considered as probable negative samples.

### 2.3 Filtering of M. grisea proteins to obtain positive-like candidate proteins

The positive-like candidate proteins of *M. grisea* were filtered out from the whole 11,054-protein sequence using transmembrane, extracellular localization, and secretory protein information. The *M. grisea* proteins are identified as transmembrane, when predicted transmembrane helices were more than one using TMHMM (Krogh et al., 2001). BUSCA (Savojardo et al., 2018) is used to locate extracellular localization. Finally, the SignalP (Bendtsen et al., 2004) predictor is used to identify secretory protein information. Using the above three tools, a total of 1,572 *M. grisea* proteins were identified as positive-like candidates. These 1,572 proteins were subjected to blast analysis against the remaining 9,482 *M. grisea* proteins having an E-value of 10^-5^ to obtain the homologs. From this analysis, a total of 4,226 homolog proteins were obtained. On the other hand, only 353 proteins were obtained in *M. grisea* using the TF database (http://ftfd.snu.ac.kr/index.php?a=view) and considered as positive samples. Thus, a cumulative total of 6,151 (1,572 + 4,226 + 353) proteins of *M. grisea* were obtained and taken as positive-like samples that might be participating in the interactions. On the other hand, the remaining 5,256 proteins were considered as negative samples that do not participate in the interactions.

Experimentally verified PPIs were collected between rice and *M. grisea* (Pellegrini et al., 1999; Jia et al., 2000; Krogh et al., 2001; Ng et al., 2003; Salwinski et al., 2004; Quevillon et al., 2005; Shoemaker and Panchenko, 2007; Wang et al., 2007; Najafabadi and Salavati, 2008; Parker et al., 2008; Ribot et al., 2008; Kumar and Nanduri, 2010; Mukhtar et al., 2011; Li et al., 2012; Maetschke et al., 2012; Mentlak et al., 2012; Park et al., 2012; Schleker et al., 2012; Simonsen et al., 2012; Meyer et al., 2013; Mosca et al., 2014; Rao et al., 2014; Sahu et al., 2014; Tully et al., 2014; Nourani et al., 2015; Li et al., 2016; Singh et al., 2016; Klopfenstein et al., 2018; Savojardo et al., 2018; Karan et al., 2019; Ma et al., 2019; Sahu et al., 2019; Loaiza et al., 2020; Lu et al., 2020; Singh et al., 2020; Wang et al., 2020; Rapposelli et al., 2021; Kumar et al., 2022; Mishra et al., 2022; Wu et al., 2015) from an exhaustive literature survey and used an independent dataset **Table 1**.

**Table 1:** List of experimental validated PPIs retrieved from literature search.

S. No.	Rice gene	Accession ID	Pathogen gene	Accession ID	Reference
1	Pita, Pi-4a	LOC-Os12g18360	AVR-Pita	MGG-15370	Jia et al. (2000)
2	OsExo70-F3	LOC-Os04g31330	AVR-Pii	MGG-08024	Fujisaki et al. (2015)
3	OsExo70-F2	LOC-Os02g30230	AVR-Pii	MGG-08024	
4	RGA-4	NCBIrefseq: XP_015619689.1	AVR-Pia	BAH23994.1	Cesari et al. (2013)
5	RGA-4	NCBIrefseq: XP_015619689.1	AVR1-Co39	UniProtKB/Swiss-Prot: Q8J180	
6	RGA-5	AGM61351.1	AVR-Pia	BAH23994.1	
7	RGA-5	AGM61351.1	AVR-Co39	UniProtKB/Swiss-Prot: Q8J180	
8	Pikh, Pi54	ALO78751.1	AVR-Pi54	MGG-03685	Devanna et al. (2014)
9	APIP6	LOC-Os05g06270	AVR-Pizt	MGG18041	Park et al. (2012)
10	Pia	AGM61350.1	AVR-Pia	BAH23994.1	Yoshida et al. (2009)
11	Pii	BAN59294.1	AVR-Pii	MGG-08024	
12	pik-m	BAH79889.1	AVR-pikm	BAP47455.1	
13	pikp-1	ADV58352.1	AVR-pik	BAH59490.1	
14	pikm-1	BAH79878.1	AVR-pik	BAH59490.1	
15	Pikh	AIY55350.1	AVR-pik	BAH59490.1	
16	Piks	AET36547.1	AVR-piks	BAH59490.1	
17	Os NADP-ME2	LOC-Os01g52500	AVR-Pii	MGG-08024	Singh et al. (2016)
18	CEBIP (BIC)	Swiss-Prot:D7UPN3.1	SLP1	NCBIrefseq: XP_003714140.1	Mentlak et al. (2012)
19	Pib	BAA76282.2	AVR-Pib	AKO62639.1	Zhang et al. (2015)
20	OsNLP1	Swiss-Prot: Q10S83.1	MONEP1	ADM07417.1	Zhang et al. (2015)
21	Pi9, pit2	LOC-Os06g17900	AVR-Pi9	MGG12655	Wu et al. 2015
22	Piz-t	ABC73398.1	AVR-Pizt	MGG18041	

### 2.4 Development of computational models to predict the PPIs in rice and M. grisea

**Figure 1:**
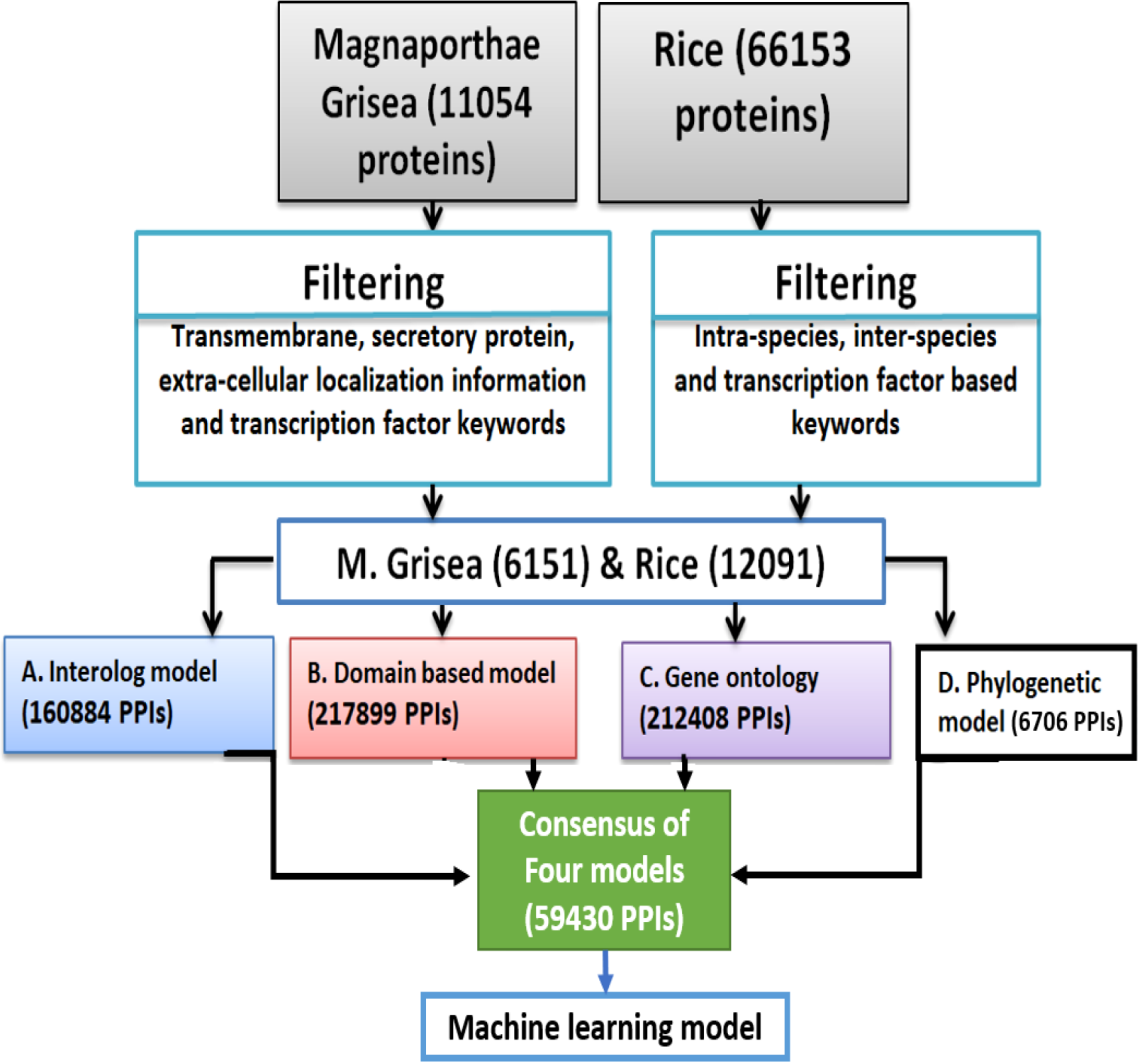
A schematic representation showing the overall prediction network for the proposed model development.

The 12,091 positive-like rice proteins as well as 6,151 positive-like *M. grisea* proteins were used for the development of computational models. In this study, PPI was determined using domain-based, interolog, GO, and phylogenetic-based models. **Figure 1** shows a schematic representation of the entire model development.

#### 2.4.1 Interolog model for PPI prediction

**Figure 2:**
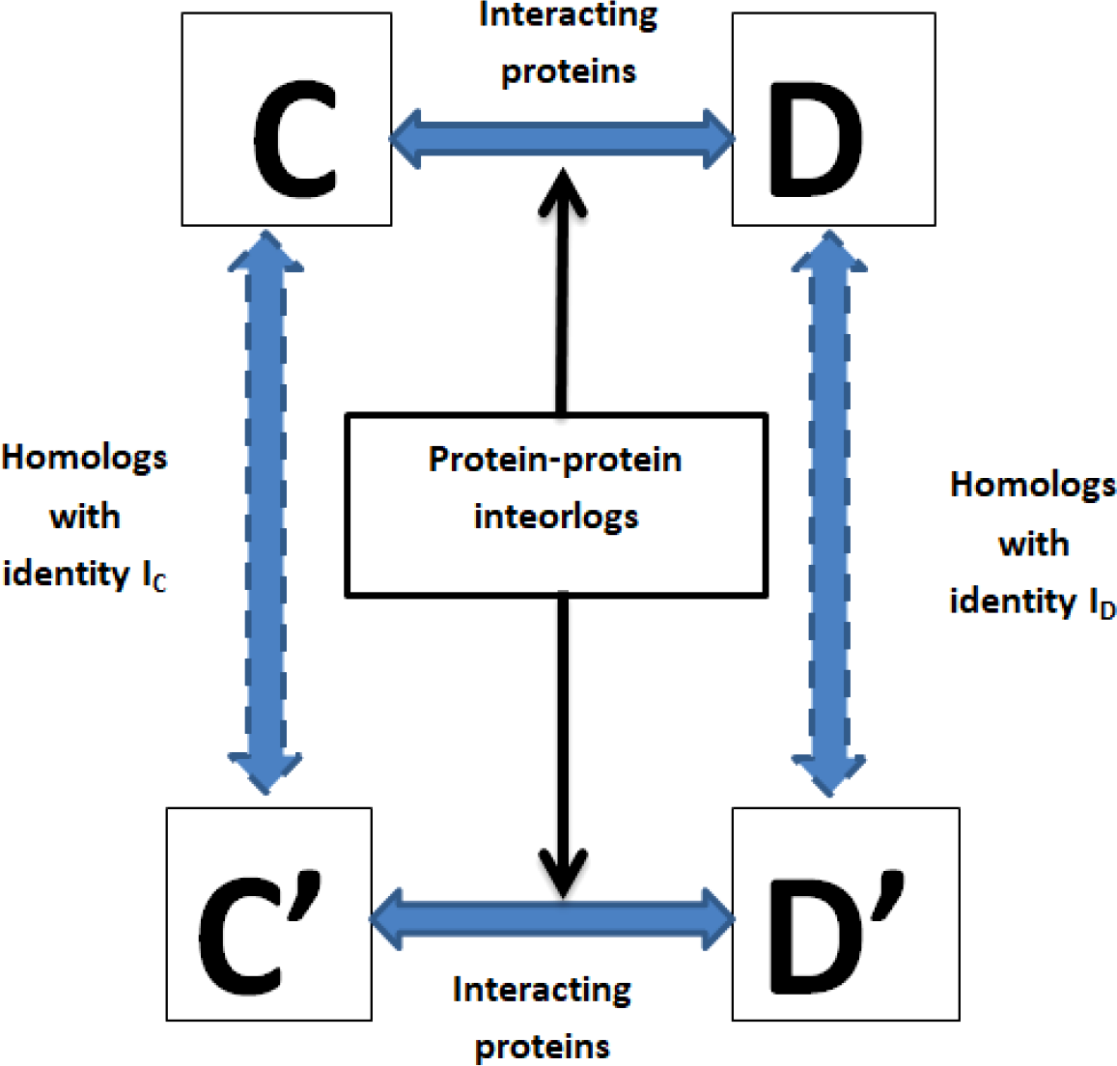
A schematic presentation of the interolog model for protein–protein interaction prediction. Here, (C, D) are two interacting proteins, and (C’–D’) are the proteins among which interaction needs to be predicted. Protein C’ is homologous to (C, D’) is homologous to (D), then (C’, D’) are likely to interact.

The interolog model relies on the similarity of the protein sequences (Savojardo et al., 2018). If an interaction between their homologous proteins happens, each protein pair between the pathogen and the host is expected to interact (Meyer et al., 2013). A schematic presentation of the interolog model is shown in **Figure 2**. Each protein of rice and *M. grisea* was subjected to BLAST analysis against host and pathogen proteins in the HPIDB (Kumar and Nanduri, 2010) database having an E-value of 10^-5^. Like the above criteria, each protein of rice and *M. grisea* was also subjected to BLAST analysis against the DIP (Salwinski et al., 2004) database. If there is an experimentally confirmed interaction with their respective homologous proteins in the DIP or HPIDB databases, it is assumed that each protein pair between *O. sativa* and *M. grisea* will interact.

#### 2.4.2 Domain-based model for PPI prediction

**Figure 3:**
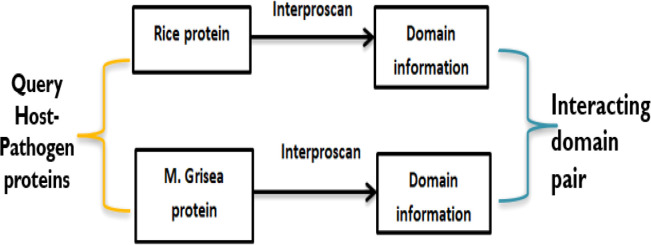
A schematic illustration showing a domain–domain method for PPIs.

To predict potential PPIs, the domain-based method uses the knowledge of information based on domain–domain interaction that has been derived from known 3D structures of proteins. Here, if two query proteins contain a pair of interacting domains, then these two proteins are the most probable candidates to interact with each other (Ng et al., 2003). A schematic presentation of the domain-based model has been given in **Figure 3**. To obtain the domains related to rice and *M. grisea*, interproscan5 (Quevillon et al., 2005) is used. Rice and *M. grisea* domains were searched against the repositories of domain–domain interaction such as Instruct (Meyer et al., 2013), Pfam (El-Gebali et al., 2019), and 3did (Mosca et al. 2014). Instruct http://instruct.yulab.org/ is a database annotated to the 3D structural resolution of protein interactome networks. Pfam https://pfam.xfam.org/ is a protein domain–domain interaction database that includes their annotation and multiple sequence alignment generated using the Hidden Markov model. The Pfam database contains 16,642 domain–domain interaction pairs. The three-dimensional interacting domains 3did database https://3did.irbbarcelona.org/index.php is a set of 3D high-resolution structural models for domain–domain interactions. The 3did is composed of 14,726 domain–domain interaction pairs. If a pair of proteins contains an interacting pair of domains from the repositories, then the pair is supposed to interact (Shoemaker and Panchenko, 2007).

#### 2.4.3 Gene ontology-based model for PPI prediction

The GO model is based on the hypothesis that proteins that interact within a cell are more likely to be in similar places or engaged in similar biological processes (Jain and Bader, 2010).

##### 2.4.3.1 GO model development:

█ The GO term related to cellular components, biological process, and molecular function is obtained for both rice and *M. grisea* protein using the GO-Blast tool.
█ Resnik’s max method is used for calculating the semantic similarity score between paired GO terms [51]. Resnik’s method uses the information content specified in Jain and Bader (2010) to compute the semantic similarity (S) between ontology terms m and n for a given set C of ancestors a and b.

$$\text{S}\left(\text{a},\text{b}\right)=\underset{\text{c}\varepsilon \text{C}}{\max}\left[-\ln\left(\text{p}\left(\text{c}\right)\right)\right]$$

**Figure 4:**
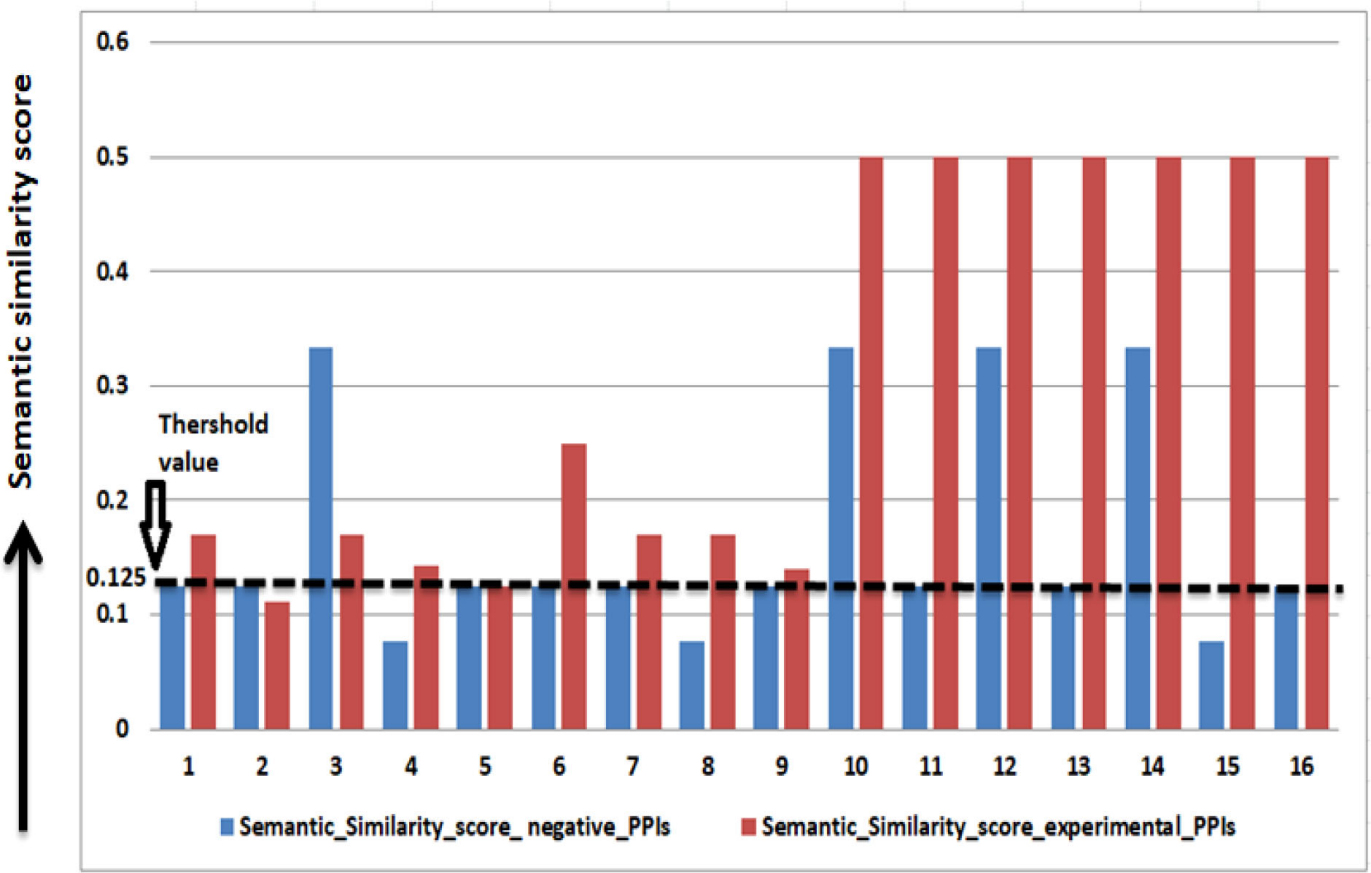
Threshold value calculation from experimentally verified PPIs.

A threshold of 0.125 on the semantic similarity is obtained from 16 experimentally verified PPIs (refer to **Table 2** to identify the interacting pair). The PPIs having a semantic similarity score of 0.125 or more than 0.125 is considered as potential PPIs (**Figure 4**).

**Table 2:** List of potential rice and *M. grisea* protein participating in multiple interactions.

Proteins	Protein name
Rice	LOC_Os02g03060.2, LOC_Os02g05480.1, LOC_Os02g05480.2, LOC_Os05g25450.1, LOC_Os02g54510.3, LOC_Os01g59360.1
*M. grisea*	MGG_06320T0, MGG_14847T0, MGG_02757T0, MGG_08689T0, MGG_04790T0, MGG_14773T0, MGG_00803T0, MGG_12821T0, MGG_01998T0, MGG_01596T0, MGG_06599T0, MGG_09960T0, MGG_06382T0

In the GO-based model, 212,408 PPIs are predicted between 4,321 rice and 753 *M. grisea* proteins.

#### 2.4.4 Phylogenetic-based model for PPI prediction

**Figure 5:**
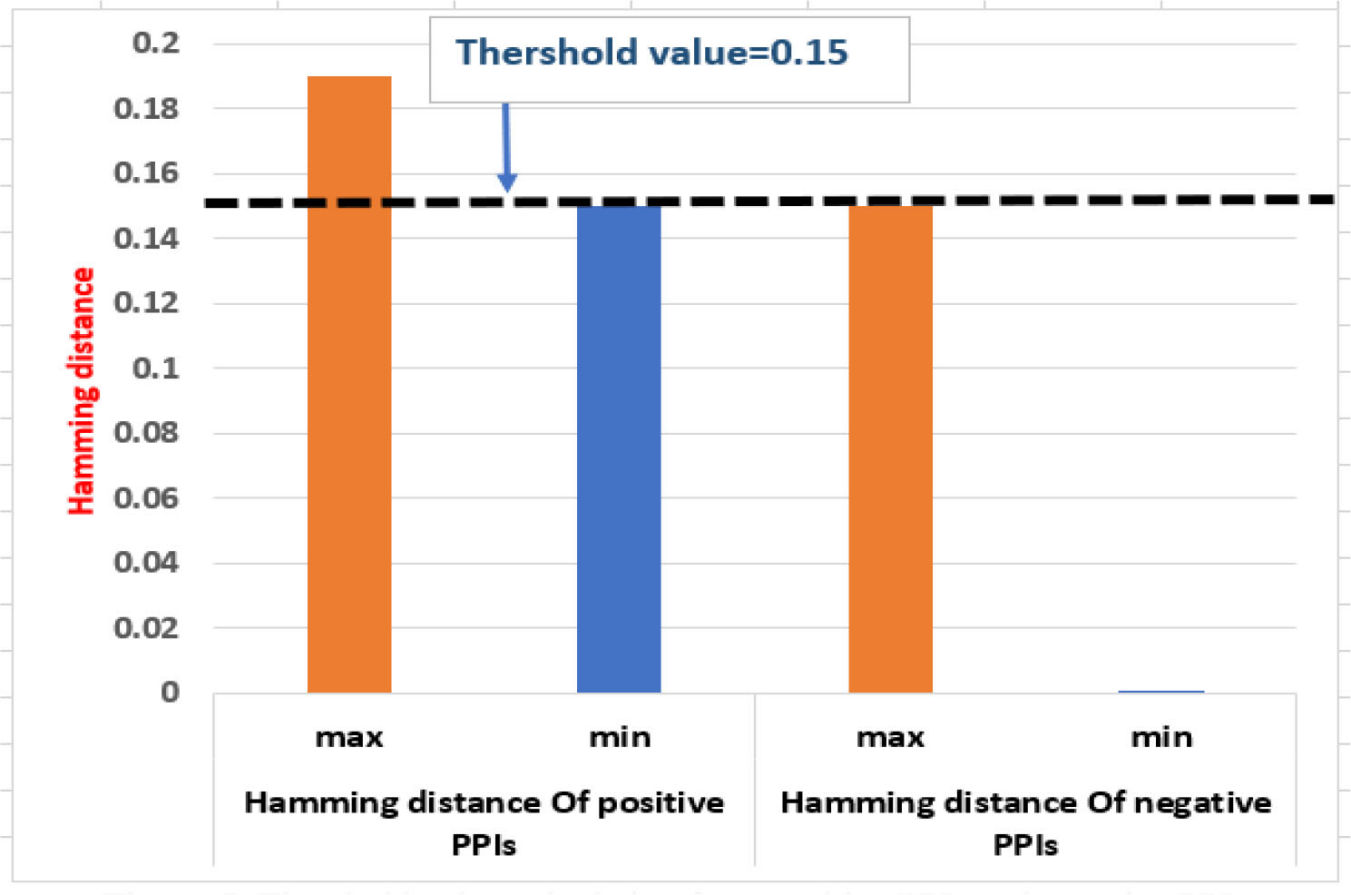
Threshold value calculation from positive PPIs and negative PPIs.

The phylogenetic profile of proteins is used for predicting the PPIs (Simonsen et al., 2012). It is based on the idea that functionally related proteins are more likely to coexist or be removed in a new species throughout evolution (Pellegrini et al., 1999). The phylogenetic profile is created by using the BLASTP (E value: 10^-5^) to recognize homologous proteins as present or absent in reference organisms. Each protein of rice and *M. grisea* was compared with the 4,045 reference organisms from UniProt using BLASTP. If any homologs are found in any reference organism, we put 1 in that place (and 0 otherwise), indicating the presence or absence of the target protein in that organism. Thus, a binary phylogenetic profile of dimension 4,145 was constructed for each protein. Subsequently, hamming distance is used to compute the similarity of the profiles. The threshold value for prediction is calculated from the positive PPIs and negative PPIs as shown in **Figure 5**.

If the similarity score is less than a threshold (0.15), the protein pairs are interacting. In the phylogenetic model, 6,706 PPIs are predicted between 160 rice and 477 *M. grisea* proteins.

#### 2.4.5 High-confidence PPIs

**Figure 6:**
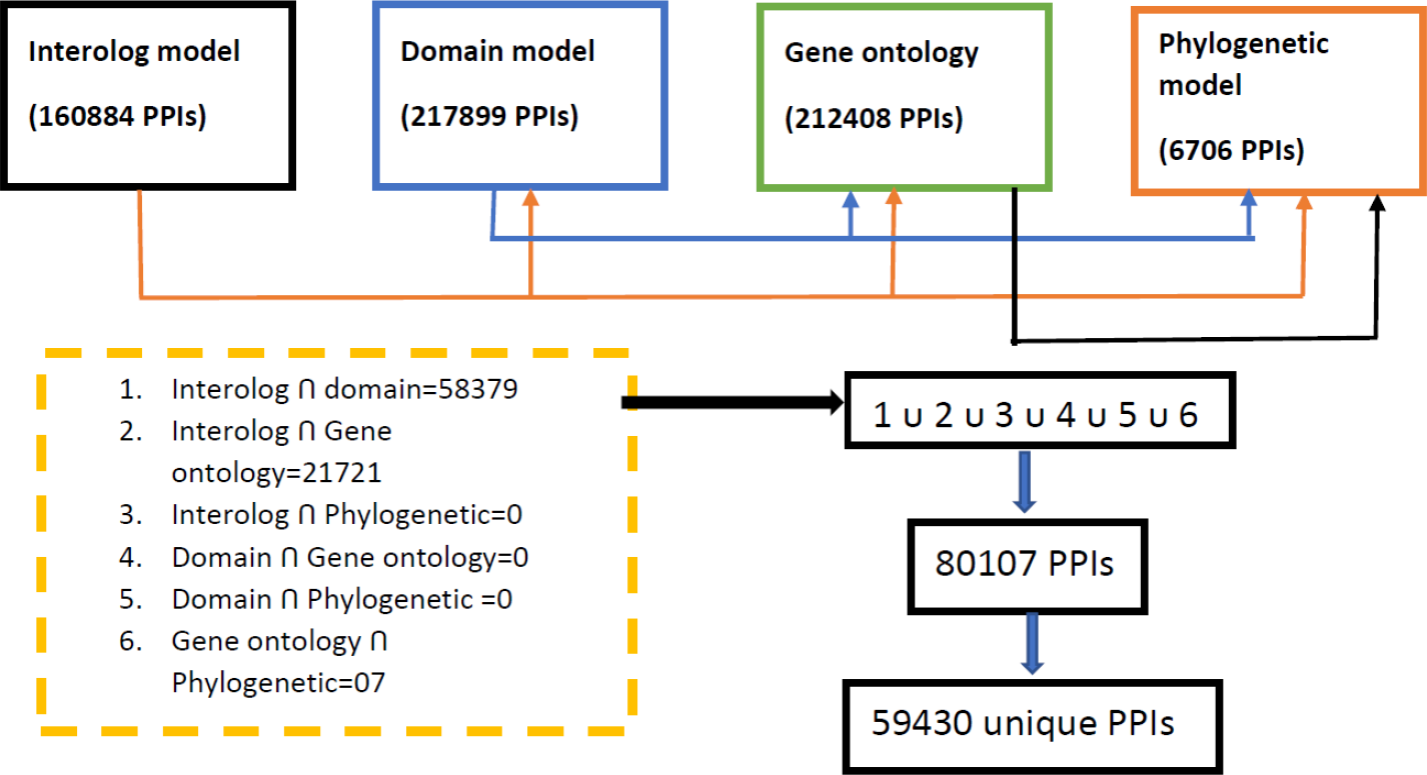
Illustration for getting the potential PPIs from the developed genomic models.

From the computational approach, it is observed that all the individual unsupervised models (domain-based, interolog, GO, and phylogenetic-based models) predict the interactions efficiently. To obtain the potential PPIs, the consensus of any of the two models was selected and then all the obtained PPIs were merged as shown in **Figure 6**.

All the consensus interactions between possible combinations of four developed models were searched and finally a total of 59,430 unique PPI pairs are obtained (**Supplementary Data Sheet 1**). The whole process of obtaining final PPIs is shown in **Figure 6**.

### 2.5 Machine learning model development

The predicted PPIs from the genomic information-based methods were further used to develop a machine learning-based model that could predict PPIs efficiently. The PPIs obtained as the consensus of four genomic models were considered as positive samples. On the other hand, an equal number of negative samples were obtained from the random pairing of probable negative candidates of rice and *M. grisea.*

#### 2.5.1 Features extraction

The widely used features, amino acid composition (AAC), and conjoint triad (CT**)** were extracted from protein sequences.

#### 2.5.2 Amino acid composition

AAC provides a 20-dimensional feature vector for each protein. For each query protein *y*, let *f*(*x_i_*) denote the frequencies of occurrence of its 20-amino acid constituent. Hence, the amino acid composition (*P_x_*) in the query protein has been represented by


(1) $$P\left(x_{i}\right)=\frac{f\left(x_{i}\right)}{\sum_{i=1}^{20}f\left(x_{i}\right)}\;\;\;\;\;\;\;\;\;i=1,2,3\ldots ...20$$

and the protein *x* in the composition space was defined as: *P*(*x*) = [*P*_1_(*x*), *P*_2_(*x*),…, *P*_20_(*x*)]. By combining their distinct AAC, each pair of host–pathogen PPI is represented by a 40-length feature vector.

#### 2.5.3 Conjoint triad

**Figure 7:**
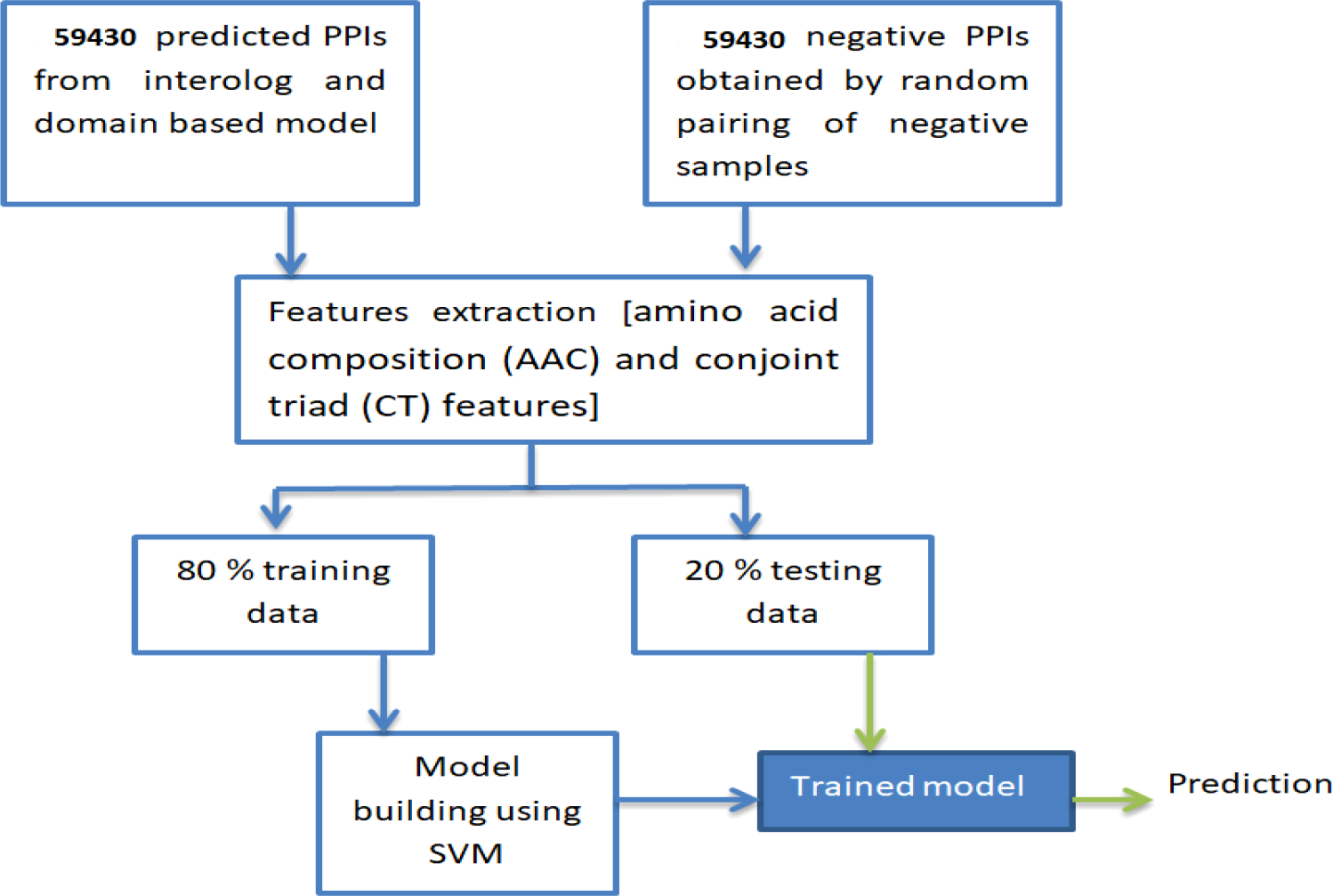
A schematic illustration of the machine learning model.

Shen et al. (Hulbert et al., 2001) first introduced the “Conjoint triad” descriptor for the protein sequence in predicting the PPIs. Based on their electrostatic and hydrophobic properties of side chain residues, the 20 native amino acids were grouped into seven classes. Each protein was described by a 343-dimensional feature vector. In the present study, to represent each PPI, the CT descriptors of the host and pathogen proteins were concatenated, resulting in the construction of 686-dimensional feature vectors. A detailed schematic experimental depiction of the constructed machine learning model is shown in **Figure 7**.

## 3 Results and discussion

**Figure 8:**
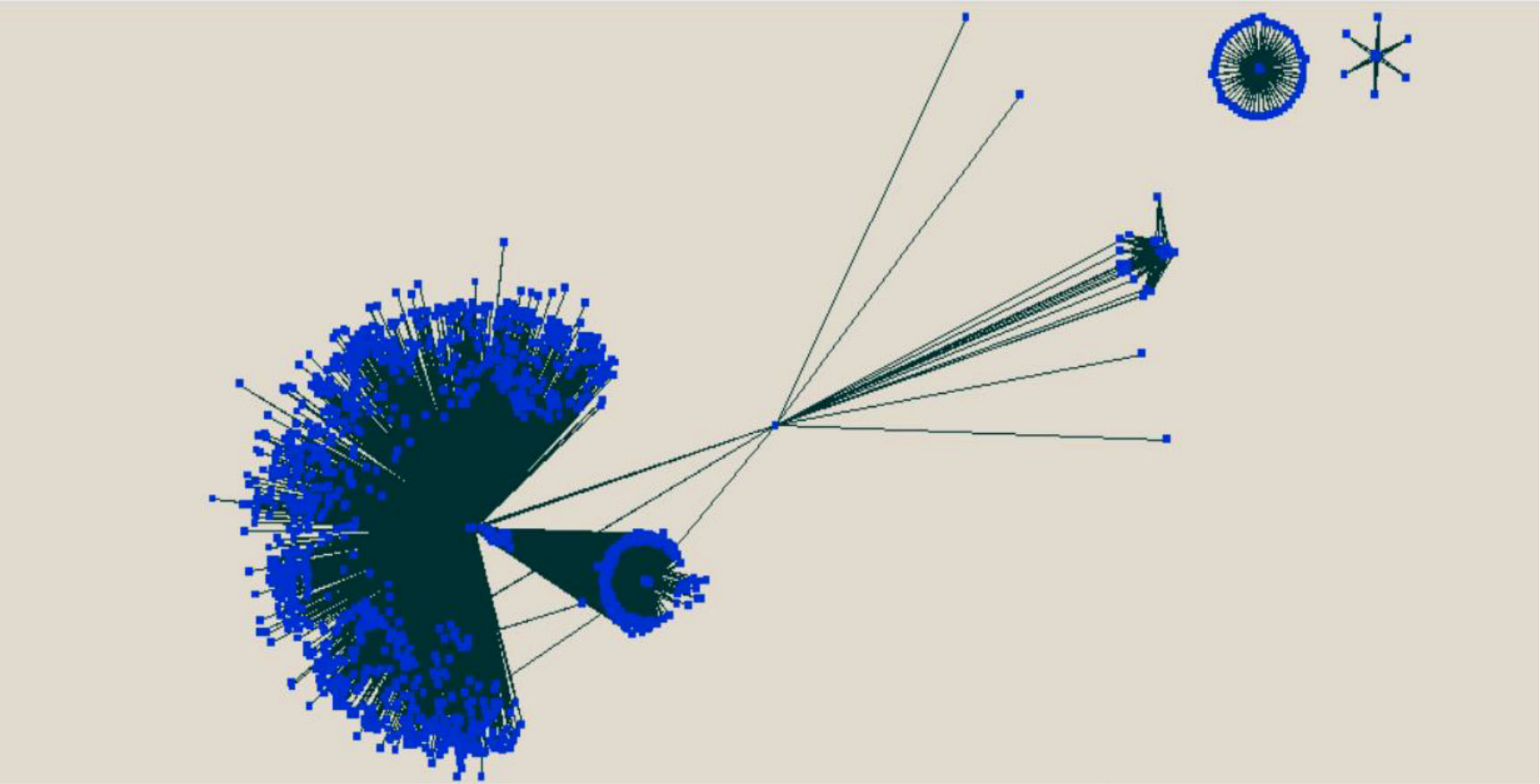
Visualization of the predicted protein–protein interaction between rice and *M. grisea* using the Cytoscape tool.

PPI has a very important role in predicting the target protein function (Rao et al., 2014). The complete protein–protein predicted network has also been visualized using the Cytoscape tool (Simonsen et al., 2012) and shown in **Figure 8**.

### 3.1 Analysis of functional enrichment of proteins involved in the interaction

The Database for Annotation Visualization and Integrated Discovery (DAVID) v6.8 is a widely used tool to verify the functional significance of the predicted host and pathogen proteins implicated in PPIs (Dennis et al., 2003). The GO enrichment analysis is conducted to observe the functional relevance of proteins used. The enrichment analysis of rice and *M. grisea* proteins has been listed in **Tables 3**, **4**, respectively. The GO terms having a *p*-value of less than 0.05 were identified to be enriched in predicted proteins.

**Table 3:** GO enrichment analysis of predicted rice proteins.

GO ID	GO Term	*p*-Value
GO:0004674	Protein serine/threonine kinase activity	1.73E-439
GO:0005524	ATP binding	1.12E-305
GO:0004672	Protein kinase activity	1.68E-247
GO:0006468	Protein phosphorylation	1.05E-186
GO:0035556	Intracellular signal transduction	9.78E-101
GO:0005886	Plasma membrane	1.11E-77
GO:0016021	Integral component of membrane	5.45E-66
GO:0018105	Peptidyl-serine phosphorylation	4.91E-57
GO:0046777	Protein autophosphorylation	3.61E-48
GO:0048544	Recognition of pollen	2.11E-45
GO:0009738	Abscisic acid-activated signaling pathway	1.01E-43
GO:0004683	Calmodulin-dependent protein kinase activity	5.69E-36
GO:0009931	Calcium-dependent protein serine/threonine kinase activity	9.39E-35
GO:0004713	Protein tyrosine kinase activity	7.01E-30
GO:0007166	Cell surface receptor signaling pathway	1.71E-29
GO:0005516	Calmodulin binding	3.95E-27
GO:0004702	Receptor signaling protein serine/threonine kinase activity	6.16E-19
GO:0006952	Defense response	1.85E-18
GO:0004707	MAP kinase activity	7.63E-18
GO:0030246	Carbohydrate binding	1.12E-16
GO:0007165	Signal transduction	1.26E-14
GO:0042626	ATPase activity, coupled to transmembrane movement of substances	4.50E-14
GO:0004693	Cyclin-dependent protein serine/threonine kinase activity	1.92E-13
GO:0008353	RNA polymerase II carboxy-terminal domain kinase activity	1.92E-13
GO:0009506	Plasmodesma	2.01E-13
GO:0005509	Calcium ion binding	8.43E-10
GO:0030247	Polysaccharide binding	1.01E-09
GO:0016055	Wnt signaling pathway	1.05E-09
GO:0006897	Endocytosis	2.03E-09
GO:0005737	Cytoplasm	3.04E-09
GO:0008360	Regulation of cell shape	4.98E-09
GO:0006950	Response to stress	7.03E-04

**Table 4:** GO enrichment analysis of predicted *M. grisea* proteins.

GO ID	GO Term	*p*-Value
GO:0005524	ATP binding	2.68E-83
GO:0004672	Protein kinase activity	7.00E-41
GO:0004674	Protein serine/threonine kinase activity	2.47E-38
GO:0018105	Peptidyl-serine phosphorylation	3.96E-08
GO:0000166	Nucleotide binding	1.94E-06
GO:0010971	Positive regulation of G2/M transition of mitotic cell cycle	3.22E-06
GO:0018107	Peptidyl-threonine phosphorylation	8.55E-06
GO:0005829	Cytosol	1.60E-05
GO:0046777	Protein autophosphorylation	2.12E-05
GO:0004693	Cyclin-dependent protein serine/threonine kinase activity	5.00E-05
GO:0005634	Nucleus	7.51E-05
GO:0032153	Cell division site	8.54E-05
GO:0051286	Cell tip	1.55E-04
GO:0030428	Cell septum	5.08E-04
GO:0004708	MAP kinase kinase activity	0.001624
GO:0004709	MAP kinase kinase kinase activity	0.001624
GO:0042787	Protein ubiquitination involved in ubiquitin-dependent protein catabolic process	0.003427
GO:0032880	Regulation of protein localization	0.003427
GO:0000307	Cyclin-dependent protein kinase holoenzyme complex	0.003436
GO:0005935	Cellular bud neck	0.00456
GO:0001403	Invasive growth in response to glucose limitation	0.010325
GO:0007124	Pseudohyphal growth	0.013999
GO:0001302	Replicative cell aging	0.013999
GO:0000196	MAPK cascade involved in cell wall organization or biogenesis	0.026172
GO:0071507	MAPK cascade involved in conjugation with cellular fusion	0.026172
GO:1990497	Regulation of cytoplasmic translation in response to stress	0.026172
GO:1902402	Signal transduction involved in mitotic DNA damage checkpoint	0.026172
GO:1990263	MAPK cascade in response to starvation	0.026172
GO:0010696	Positive regulation of spindle pole body separation	0.026172
GO:0036283	Positive regulation of transcription factor import into nucleus in response to oxidative stress	0.026172
GO:1903695	MAPK cascade involved in ascospore formation	0.026172
GO:0043332	Mating projection tip	0.036166
GO:0044878	Mitotic cytokinesis checkpoint	0.039004
GO:0071473	Cellular response to cation stress	0.039004
GO:0001402	Signal transduction involved in filamentous growth	0.039004
GO:0010515	Negative regulation of induction of conjugation with cellular fusion	0.039004
GO:0016242	Negative regulation of macroautophagy	0.039004
GO:0045860	Positive regulation of protein kinase activity	0.039004
GO:0000751	Mitotic cell cycle arrest in response to pheromone	0.039004
GO:1901196	Positive regulation of calcium-mediated signaling involved in cellular response to salt stress	0.039004
GO:0004712	Protein serine/threonine/tyrosine kinase activity	0.046542
GO:0008353	RNA polymerase II carboxy-terminal domain kinase activity	0.046542
GO:0008349	MAP kinase kinase kinase kinase activity	0.046542
GO:0038083	Peptidyl-tyrosine autophosphorylation	0.051671
GO:0031028	Septation initiation signalling	0.051671
GO:0034605	Cellular response to heat	0.051671
GO:0031134	Sister chromatid biorientation	0.051671
GO:0045835	Negative regulation of meiotic nuclear division	0.051671

It is inferred that many proteins were involved in biological processes related to metal and cadmium ions. It has been described that metal ion is required for plant defense (Fones et al., 2010; Jain and Bader, 2010). It was detected that genes are enriched with protein such as protein kinase activity, ATP binding, serine/threonine kinase activity, intracellular signal transduction, and protein phosphorylation, which are related to interaction (Jia et al., 2000; Cesari et al., 2013). Similarly, in *M. grisea*, the biological process such as ATP binding, protein kinase activity, protein serine/threonine kinase activity, and peptidyl-serine phosphorylation was enriched in the predicted genes. From the literature, it has been inferred that the identified biological process and function are closely related to the host–pathogen interaction (Pellegrini et al., 1999; Jia et al., 2000; Hulbert et al., 2001; Krogh et al., 2001; Aloy and Russell (2002); Dennis et al., 2003; Ng et al., 2003; Bendtsen et al., 2004; Glazko and Mushegian, 2004; Salwinski et al., 2004; Barker and Pagel, 2005; Dean et al., 2005; Quevillon et al., 2005; Chou and Shen, 2007; Dyer et al., 2007; Huang et al., 2007; He et al., 2008; Jaeger et al., 2008; Najafabadi and Salavati, 2008; Parker et al., 2008; Ribot et al., 2008; Fones et al., 2010; Jain and Bader, 2010; Kumar and Nanduri, 2010; Mukhtar et al., 2011; Bai et al., 2012; Braun and Gingras, 2012; Das et al., 2012; Li et al., 2012; Maetschke et al., 2012; Mentlak et al., 2012; Park et al., 2012; Schleker et al., 2012; Cesari et al., 2013; Meyer et al., 2013; Devanna et al., 2014; Mosca et al., 2014; Rao et al., 2014; Sahu et al., 2014; Fujisaki et al., 2015; Huo et al., 2015; Nourani et al., 2015; Li et al., 2016; Berkey et al., 2017; Klopfenstein et al., 2018; Savojardo et al., 2018; Ding and Daisuke, 2019; El-Gebali et al., 2019; Karan et al., 2019; Ma et al., 2019; Sahu et al., 2019; Loaiza et al., 2020; Lu et al., 2020; Farooq et al., 2021; Rapposelli et al., 2021; Kumar et al., 2022; Mishra et al., 2022; Shoemaker and Panchenko, 2007).

### 3.2 Subcellular localization of rice proteins

**Figure 9:**
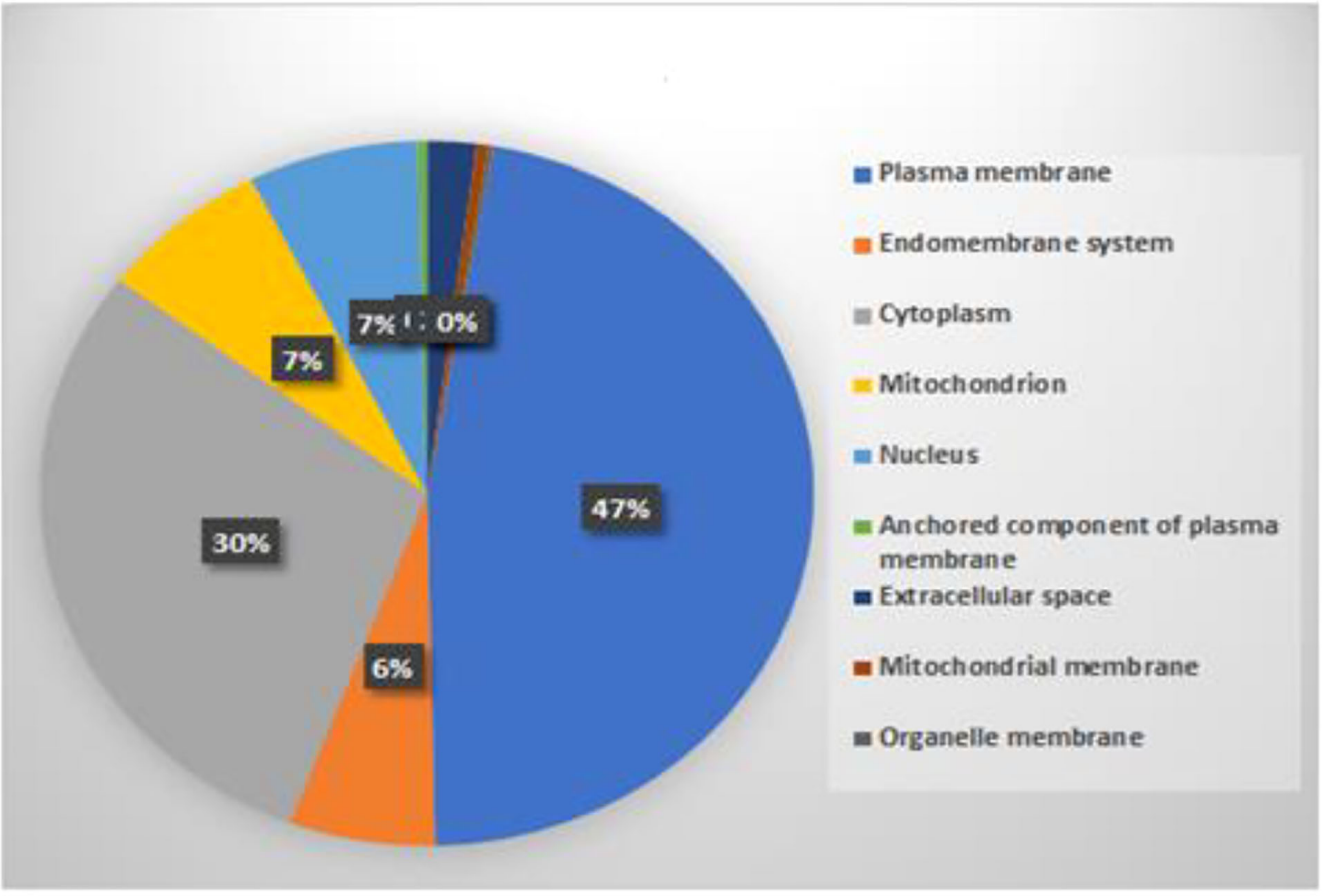
Distribution of predicted rice proteins in subcellular localization.

To check the location of predicted interacting rice proteins, their subcellular localization was extracted using BUSCA (Savojardo et al., 2018). Results revealed that subcellular localization of predicted proteins was distributed in the cytoplasm, plasma membrane, nucleus, mitochondria, extracellular space, and endomembrane by 46%, 29%, 7%, 7%, 2%, and 6%, respectively, as shown in **Figure 9**. The subcellular localization of gene product with the site of their interactions has already been reported (Bai et al., 2012; Das et al., 2012; Berkey et al., 2017; Singh et al., 2020). A detailed list of cloned blast resistance gene *Pi54* overexpressed in rice for understanding its cellular and subcellular localization and response to different pathogens has been reported (Singh et al., 2020). Due to the advancement in rapid genome sequencing techniques, annotation and subcellular localization of uncharacterized plant proteins are very important. Considering this important challenge, classifiers, namely, Plant-PLoc and Plant-mSubP, have been developed and reported for large-scale subcellular location prediction for plant proteins (Chou and Shen, 2007; Sahu et al., 2019). Our predicted result infers that a major interaction occurs in the plasma membrane and cytoplasm, which is in line with the literature.

### 3.3 Identified hub protein in rice and *M. grisea*

**Figure 10:**
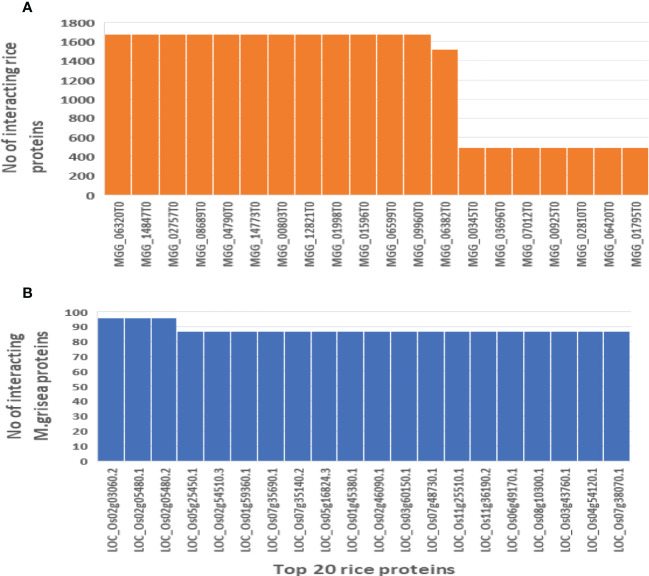
Hub nodes in the rice and *M. grisea* protein–protein interaction network. **(A)** Number of interactions in top 20 rice hub proteins and **(B)** number of interactions in top 20 *M. grisea* hub proteins.

In biological networks, PPI hubs have a significant role in the pathogenicity mechanism. The hub proteins that have many interacting partners were identified. The top 20 hub proteins with their interacting partners are shown in **Figure 10**.

These identified hub proteins might be used for drug target design. From **Table 2**, it is inferred that rice proteins like LOC_Os02g03060.2, LOC_Os02g05480.1, LOC_Os02g05480.2, LOC_Os05g25450.1, LOC_Os02g54510.3, and LOC_Os01g59360.1 were involved in more than 80 interactions with *M. grisea*. On the other hand, in case of *M. grisea* out of 126 proteins, 49 were involved in multiple interactions. The top 11 *M. grisea* proteins are MGG_06320T0, MGG_14847T0, MGG_02757T0, MGG_08689T0, MGG_04790T0, MGG_14773T0, MGG_00803T0, MGG_12821T0, MGG_01998T0, MGG_01596T0, and MGG_06599T0 (**Table 2**). These *M. grisea* pathogen proteins are interacting with more than 1,600 rice proteins, indicating that these genes are important for interaction and pathogenesis.

### 3.4 Development of the machine learning model

#### 3.4.1 Training/testing schema

To develop the machine learning model, a total of 59,430 computationally predicted PPIs have been used as a positive dataset whereas a negative dataset was prepared by random pairing of the negative candidate proteins generated from a filtered non-interacting sequence of rice and *M. grisea* as described in *Section 2*. Here, a fivefold cross-validation scheme was used for model development (Karan et al., 2019; Li et al., 2016). Training accuracies of 95% and 99% were obtained with AAC and CT features, respectively (Sahu et al., 2014). SVM-based testing performance for AAC indicated its accuracy, sensitivity, and specificity as 88%, 89%, and 86%, respectively. On the other hand, SVM-based testing performance for CT provides an accuracy, sensitivity, and specificity of 89%, 84%, and 93%, respectively (**Table 5**). Furthermore, the model was assessed with 22 experimentally verified PPIs as an independent test set. Importantly, 21 out of 22 samples were predicted as positive based on CT features (**Table 5**).

**Table 5:** SVM-based analysis result for amino acid composition and conjoint triad features.

1	**SVM-based testing performance (kernel = RBF, Gamma = 0.4, C = 1,000) in test dataset**			
	Features	Accuracy (%)	Sensitivity (%)	Specificity (%)
	Amino acid composition	88	89	86
	Conjoint triad	89	84	93
2	SVM-based prediction result of experimental verified dataset (independent dataset)			
	Features	Total no. of PPIs	No. of true positives	Accuracy (%)
	Amino acid composition	22	17	77
	Conjoint triad	22	21	95

#### 3.4.2 Testing with other host–pathogen systems

The predicted model was further assessed with various host–pathogen systems to evaluate its reliability. Datasets of animal with *Bacillus* (set 1), hepatitis C virus (set 2), measles virus (set 3), *Yersinia* (set 4), and herpes virus (set 5) were used for analysis. While the dataset of the *Arabidopsis thaliana* plant was used for analysis with *Pseudomonas syringae* (set 6). The animal pathogen database was extracted from HPIDB 2.0 version while the *Arabidopsis thaliana–Pseudomonas syringae* database was extracted from Mukhtar et al. (2011) and Tully et al. (2014). The false-positive results are shown in **Table 6**. AAC feature analysis revealed that the percentage of false-positive values for sets 1 to 5 was 21%, 0.02%, 2%, 14%, and 8%, respectively. The percentage of false-positive value between *Arabidopsis thaliana* with *Pseudomonas syringae* (set) was 41%. On the other hand, CT feature analysis revealed that the percentage of false-positive values for sets 1 to 6 was 12%, 14%, 11%, 9%, 6%, and 5%, respectively. The percentage of false-positive value between *Arabidopsis thaliana* with *Pseudomonas syringae* was 15%. From **Table 6**, it is noticed that the prediction accuracy of average FP positive is approximately 14.33% in case of AAC and 11.6% using CT features. This revealed that the model was specific to rice and *M. grisea*.

**Table 6:** Comparative performance of amino acid composition and conjoint triad features with other host–pathogen systems.

			Amino acid composition		Conjoint triad	
Sl no.	Independent host–pathogen system	Total PPIs	# of False positives	False positive (%)	# of False positives	False positive (%)
1.	Set 1	3,090	660	21	370	12
2.	Set 2	3,295	9	0.02	475	14
3.	Set 3	994	24	2	109	11
4.	Set 4	4,296	600	14	381	9
5.	Set 5	9,152	847	8	577	6
6.	Set 6	166	68	41	25	15
**Average performance (%)**				14.33		11.6

The machine learning model performance was compared with a similar study reported previously by Ma et al. (2019) who have reported 532 potential PPIs using interolog and domain-based methods. The similar number of negative PPIs are extracted from the negative datasets obtained by the random pairing of filtered rice and *M. grisea* protein sequence. A machine learning model using support vector machine is developed using 532 positive and 532 negative PPIs. The obtained machine learning model was tested with 22 experimental datasets (**Table 1**). A total of 17 PPIs are predicted using AAC features with an accuracy of 77%. Also, the developed machine model was tested with CT features, and it provides 95% accuracy (**Table 7**).

**Table 7:** SVM-based comparative prediction result of amino acid composition and conjoint triad features in the experimental verified dataset with the Ma et al. (2019) model and our proposed model.

	Testing with the model obtained from PPIs from Ma et al. (2019)			Testing with the model obtained from PPIs from our proposed method		
Features	Total no of PPIs	# of True positives	Accuracy (%)	Total no. of PPIs	# of True positives	Accuracy (%)
Amino acid composition	22	17	77	22	17	77
Conjoint triad	22	1	4	22	21	95

The main difference between the work of Ma et al. (2019) and our proposed work was in the filtering process involved. Ma et al. removed the PPIs with rice fungus proteins annotated with non-membrane and non-secreted ones from the intersection potential PPIs obtained from interolog and domain-based models. In contrast, in the present study, both rice and fungus proteins were first filtered out using a well-analyzed filtering process. The interolog and domain-based model was employed on the filtered database. The limitation of Ma et al.’s work was that the developed machine learning model was not tested with an independent dataset and other host–pathogen systems. Zheng et al. (2021) presented a computer methodology for structurally based plant–pathogen PPI prediction in rice and fungus.

PPI has a key role in predicting the functions of uncharacterized protein as well as in determining its role in the phenotypic responses. PPIs are involved in controlling the various biological processes like cell-to-cell interactions as well as metabolic and developmental processes (Braun and Gingras, 2012). Reports describing the PPIs in drug discovery (Rapposelli et al., 2021), the development of PPI modulators (Lu et al., 2020), and PPI applications in virus–host study (Farooq et al., 2021) have been published. Also, a rice protein interaction network revealing high centrality nodes and candidate pathogen effector targets (Mishra et al., 2022) and another pipeline of integrating transcriptome and interactome for elucidating central nodes in host–pathogen interactions (Kumar et al., 2022) have been published. The present study provides a genome-wide PPI between rice and *M. grisea.* Furthermore, it is accurate and computationally inexpensive because of its filtering process prior to computational model development. Furthermore, a validation study on predicted PPI subcellular localization may also be carried out in the future.

## 4 Conclusion

In this study, several computational models are developed using the interolog, domain, GO, and phylogenetic information to predict the PPI between rice and *M. grisea* in a genome. A total of 59,430 highly confident PPIs are predicted between 1,801 rice proteins and 135 *M. grisea* proteins. The GO enrichment analysis shows that the predicted proteins are involved in interactions related to functionalities. Furthermore, to assess the effectiveness of predicted PPIs, a machine learning model based on support vector machine is developed. Based on the fivefold cross-validation test, better accuracy is obtained using AAC and CT features of protein sequence. Furthermore, the proposed model was tested on 22 experimentally identified PPIs between rice and *M. grisea* in an independent test that resulted in the prediction of 21 PPIs as positive using CT features. The reliability of the proposed model is also checked for PPIs on various host–pathogen systems. The proposed model predicted a lower number of PPIs as positive, inferring that the method is specific to rice and *M. grisea*. The predicted PPIs could be a useful resource for further studies on the rice–*M. grisea* interaction mechanism.

## Data availability statement

The original contributions presented in the study are included in the article/**Supplementary Material**. Further inquiries can be directed to the corresponding author.

## Author contributions

BK: Methodology, Software, Writing – original draft; SM: Validation, Investigation, Writing - review & editing. SS: Methodology, review & editing; DP: Validation, Investigation; SC: review & editing. All authors contributed to the article and approved the submitted version.
