# Supplementary material for: Computational models for prediction of protein–protein interaction in rice and Magnaporthe grisea
Source: Front Plant Sci. 2023 Feb 1;13:1046209. doi: 10.3389/fpls.2022.1046209 (PMC9929577; doi:10.3389/fpls.2022.1046209)
Supplement: Supplementary file 2 [file Table_1.docx]

Table 1: List of keywords that have been used for searching the intraspecies interaction from the databases

| **List of keywords** | | | | |
| --- | --- | --- | --- | --- |
| Binding | G-protein | Ligand-grated | Membrane | Folding |
| Disease | Defense | Interaction | Resistant | Kinase |
| Receptor | Receptor-like | Peripheral | Cysteine | Leucine, Coiled |
| Microbe-associated | Cell-cell communication | Ion channel | Transmembrane | GPCR |
| Enzyme-linked | Extracellular | Adenylyl cyclase | Adenylate | Phospholipase |
| PDZ domain | ATP-binding | Defensin | Resistance |  |
| Effector | Cell membrane, |  |  |  |

Table 2: List of keywords that have been used for searching the interspecies interaction from the databases

| **List of keywords** | | | | |
| --- | --- | --- | --- | --- |
| DNA binding | mRNA caping | Ubiquitination | Ubiquitin | RNA binding |
| Oxidoreductase | Signaling | Auxin signaling | Calmodulin |  |
| Germination | Transmembrane | DNA rapair | Vesicle | Cytoskeleton |
| Water dikinase | Brassinosteroid | Phosphoprotein | Nitrogen | Nucleosome |
| Transcription | DNA polymerase | Potassium | Root hair | Ribonucleoprotein |
| mRNA splicing | Kinase | Transport | Polymerase | Aromatic |

Table 3: List of keywords that have been used for searching the interaction from the *Oryzasativa* transcription factor database

| **List of keywords** | | | | |
| --- | --- | --- | --- | --- |
| HRT-like | C3H | LSD | LFY | LBD |
| Trihelix | C2H2 | CO-like | CAMTA | ERF |
| GeBP | DBB | EIL | E2F/DP | GRF |
| HSF | FAR1 | GRAS | GATA | G2-like |
| MIKC_MADS | ARR-B | HD-ZIP | HB-other | HB-PHD |
| SBP | ARF | CPP | B3 | BBR-BPC |
| M-type MADS | Dof | NAC | MYB related | MYB |
| NF-X1 | NF-YA | Nin-like | NF-YC | VOZ |
| RAV | S1Fa-like | STAT | SRS | BES1 |
| TALE | TCP | WOX | AP2 | bHLH |
| ZF-HD | Whirly | YABBY | WRKY | NF-YB |
